# Impact of Community Mass Treatment with Azithromycin for Trachoma Elimination on the Prevalence of Yaws

**DOI:** 10.1371/journal.pntd.0003988

**Published:** 2015-08-04

**Authors:** Michael Marks, Ventis Vahi, Oliver Sokana, Kai-Hua Chi, Elliot Puiahi, Georgina Kilua, Allan Pillay, Tenneth Dalipanda, Christian Bottomley, Anthony W. Solomon, David C. Mabey

**Affiliations:** 1 Clinical Research Department, Faculty of Infectious and Tropical Diseases, London School of Hygiene and Tropical Medicine, London, United Kingdom; 2 The Hospital for Tropical Diseases, Mortimer Market Centre, London, United Kingdom; 3 Ministry of Health and Medical Services, Honiara, Solomon Islands; 4 Molecular Diagnostics and Typing Laboratory, Laboratory Reference and Research Branch, Division of Sexually Transmitted Disease Prevention (DSTDP), Centers for Disease Control and Prevention, Atlanta, Georgia, United States of America; 5 World Health Organization, Western Pacific Region Office, Honiara, Solomon Islands; 6 Department of Infectious Diseases Epidemiology, London School of Hygiene and Tropical Medicine, London, United Kingdom; Kwame Nkrumah University of Science and Technology (KNUST) School of Medical Sciences, GHANA

## Abstract

**Background:**

Community mass treatment with 30mg/kg azithromycin is central to the new WHO strategy for eradicating yaws. Both yaws and trachoma— which is earmarked for elimination by 2020 using a strategy that includes mass treatment with 20mg/kg azithromycin—are endemic in the Pacific, raising the possibility of an integrated approach to disease control. Community mass treatment with azithromycin for trachoma elimination was conducted in the Solomon Islands in 2014.

**Methods:**

We conducted a study to assess the impact of mass treatment with 20mg/kg azithromycin on yaws. We examined children aged 5-14 years and took blood and lesion samples for yaws diagnosis.

**Results:**

We recruited 897 children, 6 months after mass treatment. There were no cases of active yaws. Serological evidence of current infection was found in 3.6% (95% CI= 2.5-5.0%). This differed significantly between individuals who had and had not received azithromycin (2.8% vs 6.5%, p=0.015); the prevalence of positive serology in 5-14 year-olds had been 21.7% (95% CI=14.6%-30.9%) 6 months prior to mass treatment. Not receiving azithromycin was associated with an odds of 3.9 for infection (p=0.001). National figures showed a 57% reduction in reported cases of yaws following mass treatment.

**Discussion:**

Following a single round of treatment we did not identify any cases of active yaws in a previously endemic population. We found a significant reduction in latent infection. Our data support expansion of the WHO eradication strategy and suggest an integrated approach to the control of yaws and trachoma in the Pacific may be viable.

## Author Summary

Yaws is a bacterial infection closely related to syphilis. The WHO has launched a worldwide campaign to eradicate yaws by 2020. This strategy relies on mass treatment of the whole community with the antibiotic azithromycin. Mass treatment with the same antibiotic is also recommended by WHO to treat the eye condition trachoma but the dose used for this is lower. In this study we assessed the impact of a trachoma control programme in the Solomon Islands on yaws. Following a single round of mass treatment the number of yaws cases fell significantly compared to before treatment. We also saw fewer new cases of yaws being reported to the Ministry of Health. Our findings suggest that mass treatment with the lower dose of azithromycin is also effective as a treatment for yaws. In countries where both yaws and trachoma are found it may be possible to develop an integrated strategy for both conditions.

## Introduction

Yaws, caused by *Treponema pallidum* subsp *pertenue*, is a non-venereal infection closely related to syphilis that predominantly affects children living in remote, rural communities in tropical countries[1]. Infection manifests as lesions of the skin, bone and cartilage and, untreated, may progress to destructive tertiary lesions[2]. Yaws was once widespread throughout the tropics. Previous yaws control efforts in the middle of the twentieth century were based on treatment with injectable long-acting penicillin[3], and resulted in significant reductions in the burden of disease worldwide[4]. Despite these initial successes, the disease subsequently rebounded in a number of countries and it is currently thought to be endemic in at least 12 countries across West Africa, South East Asia and the Pacific[1].

In 2012, treatment with azithromycin was shown to be highly effective for yaws[5], and community mass treatment became the foundation of the new WHO Morges yaws eradication strategy[6]. Azithromycin has a number of advantageous characteristics as a mass treatment agent, including oral route of administration, long tissue half-life, and an acceptable side-effect profile.

Community mass treatment with azithromycin is also central to the control of trachoma[7], but the recommended dose used in trachoma control (20mg/kg, max 1g) is lower than that recommended for yaws (30mg/kg, max 2g). The International Task Force for Disease Eradication highlighted the need to investigate the effect of lower dose azithromycin for the treatment of yaws, and the possibility of synergies with trachoma control programmes in countries where the two diseases are co-endemic. In some areas of Ghana in which azithromycin mass drug administration was previously used for trachoma control, yaws is currently undetectable[8], supporting the hypothesis that lower dose azithromycin may be effective.

Unexpectedly, several recent studies have demonstrated that *Haemophilus ducreyi*[9,10] is a common cause of non-genital ulcerative skin lesions in children in yaws endemic communities. This is a finding which can present difficulties for clinical case identification. Community perceptions of the value of mass treatment campaigns may be affected by the impact of azithromycin on other common skin infections. Genital strains of *H*. *ducreyi* are responsive to azithromycin[11], so it is possible that mass treatment with azithromycin may have a synergistic benefit on non-yaws ulcerative skin lesions in these communities.

Both yaws and trachoma are endemic in the Solomon Islands[12], which routinely reports the third highest number of cases of yaws among all countries worldwide[13]. In 2014, the Solomon Islands Ministry of Health and Medical Services (MHMS) undertook community mass treatment with azithromycin as part of the SAFE strategy for trachoma elimination. We performed a prospective study in the Western Province of the Solomon Islands to assess the impact of azithromycin used against trachoma on the prevalence of active and latent yaws.

## Methods

As previously described [12], in September and October 2013, we conducted a pre-mass treatment survey in Western and Choiseul Provinces of the Solomon Islands, and documented a high prevalence of active and latent yaws. In mid 2014, mass antibiotic treatment was undertaken for trachoma by the Ministry of Health, in Western Province only, Choiseul Province not qualifying for mass treatment on the basis of a lower prevalence of active trachoma. Azithromycin was administered at 20mg/kg (max 1g) with dose determined by body weight, measured using analogue scales. Due to the death of a local religious leader in June 2014, some communities in the province were in mourning and did not receive treatment with azithromycin. For the purposes of this study, we randomly selected a subset of Western Province communities known to have received treatment with azithromycin. At each household, we collected data on number of residents and whether no, some, or all members of the household had received treatment with azithromycin as part of the mass treatment campaign for trachoma. We enrolled children aged 5–14 years for assessment, collecting individual level data on age, gender, the presence or absence of clinical signs and symptoms of yaws, yaws treatment history, and whether the individual reported having received treatment with azithromycin. We categorized skin lesions using the WHO yaws pictorial guide[14]. All data were entered directly into Android smartphones using the ODK software package[15].

Venepuncture was performed and a serum sample collected from each individual. In individuals with ulcerative or papillomatous skin lesions, we also collected a swab sample of lesion exudate. Exudate was transferred to a FTA Elute Micro Card (GE Healthcare, Buckinghamshire, UK) using three firm side-to-side passes of the swab across the card. We placed each card in its own re-sealable plastic packet with a sachet of desiccant. All samples were transferred to the National Referral Hospital, Honiara, where they were frozen at -20°C, and shipped on dry ice to the London School of Hygiene & Tropical Medicine (LSHTM), UK and the Centers for Disease Control and Prevention (CDC), USA for diagnostic testing.

### Laboratory testing

Serum samples were tested at LSHTM with the *Treponema pallidum* particle agglutination test (TPPA, Mast Diagnostics, Merseyside UK). On samples that were TPPA-positive, a quantitative plasma reagin test (RPR, Deben Diagnostics, Ipswich, UK) was performed. Lesion swabs were tested at the CDC using a multiplex real-time (RT) PCR for the identification of *Treponema pallidum* sub-species DNA[16]. If the *T pallidum* PCR was positive, we intended to use a second multiplex RT PCR to detect mutations in the 23S rRNA gene associated with azithromycin resistance. Regardless of the result of the *T*. *pallidum* PCR, we performed an additional duplex RT PCR for the detection of *Haemophilus ducreyi* and *Mycobacterium ulcerans* DNA[9]. All laboratory testing was performed by individuals masked to the clinical findings.

### Routine reporting of yaws incidence data

Suspected cases of yaws are reported via the MHMS Health Information System. We extracted data on the number of cases of yaws seen, per month, across all clinics in the Western Province of the Solomon Islands during the period 2011 to 2014 to allow an assessment of the impact of community mass treatment on the incidence of disease presentation.

### Statistical analysis

A positive TPPA was taken as evidence of previous or current infection. Individuals with clinical signs of yaws, a positive TPPA and an RPR titre of ≥1:4 (dual-seropositivity) were considered to have active yaws. Individuals without clinical signs of yaws and with a positive TPPA and an RPR titre of ≥1:4 were considered to have latent yaws. An RPR titre of ≥1:16 was considered to be a high-titre positive. We classified household size as ≤5 or >5 residents, 5 householders being the national average according to the most recent census[17]. Household treatment with azithromycin was categorized as complete, incomplete (at least 1 individual not treated) or none. The prevalence of active and latent yaws was compared between individuals who had and had not received treatment with azithromycin. Multivariable logistic regression was used to estimate unadjusted and adjusted odds ratios (ORs) for factors associated with both TPPA- and dual-seropositivity. Robust standard errors were used to calculate all confidence intervals (CIs) and P values, to account for village-level clustering[18]. The impact of mass treatment on cases reported to the MHMS was analysed by fitting a linear regression model to the time series on incident yaws cases, controlling for known seasonal variations and trend in yaws incidence. To account for autocorrelation, the error in the model was assumed to follow an autoregressive process, with a lag of one. All analyses were performed using Stata 13.1 (Statacorp, Texas).

### Sample size

Our pre-mass drug administration (MDA) survey had shown that the prevalence of dual-seropositivity in these communities was approximately 20%[12]. Assuming that treatment with azithromycin is 90% effective, the prevalence in people who receive treatment would be anticipated to be approximately 2% post treatment. The prevalence of yaws in untreated individuals was also predicted to fall due to reduced community transmission, although there were no data to guide the likely magnitude of this effect. Assuming, conservatively, that prevalence amongst untreated individuals would fall by 25%, 72 individuals receiving azithromycin and 72 individuals who did not receive azithromycin would have 90% power to detect a difference in the prevalence of yaws. Given anticipated community coverage of 90%, a total survey sample of 720 individuals would therefore be required.

### Ethical approval

Written informed consent was obtained from each participating child’s parent or guardian by a member of staff fluent in the local dialect. Assent was obtained from all children. Ethical approval for the study was granted by the ethics committees of the Solomon Islands MHMS and the LSHTM (6358).

## Results

We enrolled 897 children from 441 households in 11 communities. The median age of children was 9 years, and 466 (52%) were male. 717 children (80%) reported having been treated with azithromycin as part of the trachoma control programme (Table 1) (S1 File).

**Table 1:** Demographics of study subjects.

Number of children		897
Number of households		441
Household size (Median, IQR)		6 (4–7)
Household MDA Coverage	Complete	59%
	Incomplete	28%
	None	13%
Age (Median, IQR)		9 (7–12)
Male		466 (52%)
Received treatment with azithromycin		717 (80%)
Reported treatment for yaws in last year		93 (10%)

Two hundred and thirty seven children (26%) had a clinically apparent skin lesion. Twenty-eight children (3.1%) had a skin lesion clinically consistent with yaws. Lesions were more common in individuals who had not received MDA, but this difference was not statistically significant. (4.9% vs 2.6%, p = 0.101). No individual with a skin lesion consistent with active yaws had dual-positive serology. Bone swelling consistent with secondary yaws was rare, occurring in only 4 subjects (0.5%). Sixty children (6.7%) had skin lesions consistent with healed yaws. Other skin lesions including ringworm and bacterial infections were common (158 children, 17.6%).

**Fig 1:**
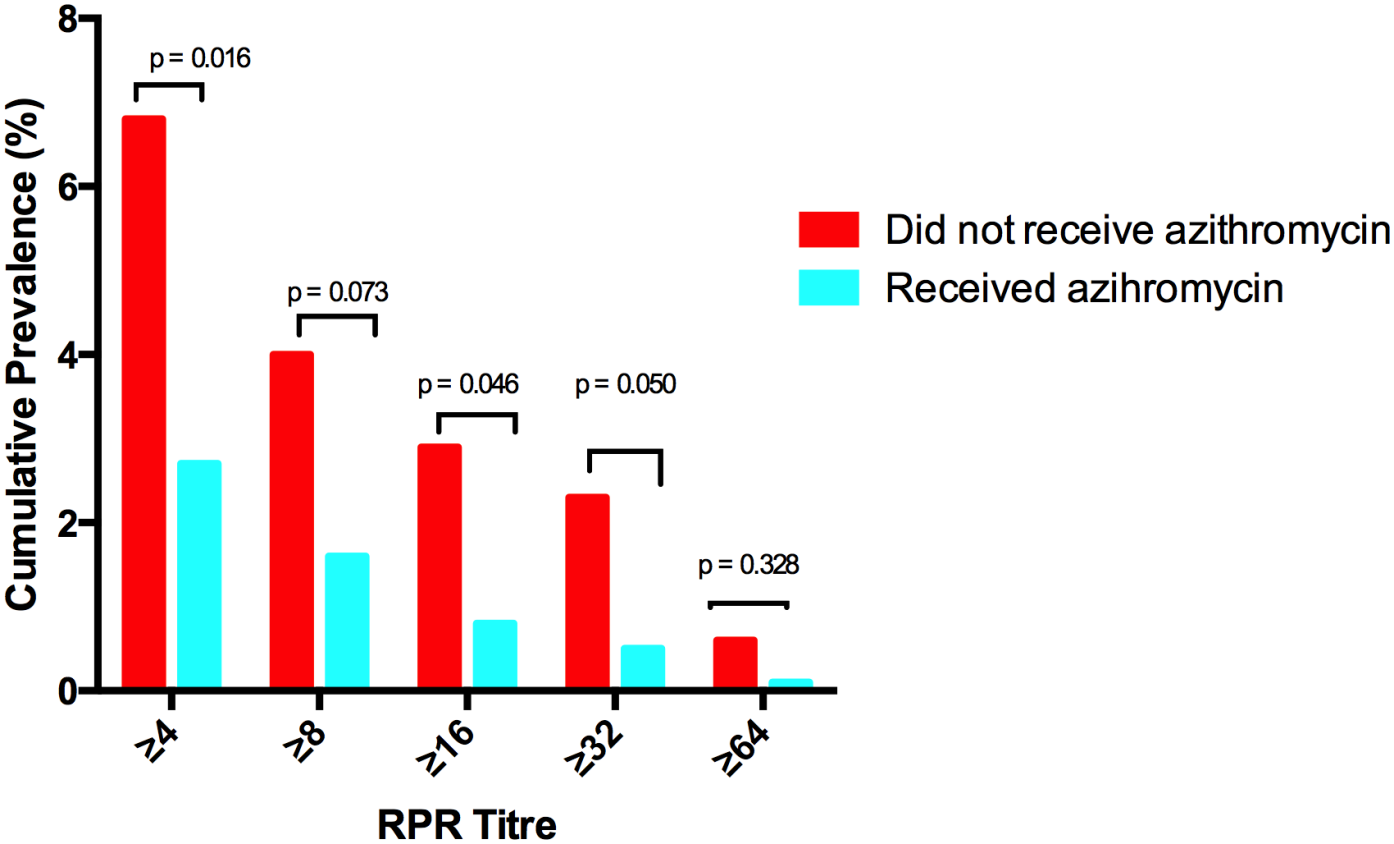
Prevalence of dual sero-positivity in individuals who did and did not receive treatment with azithromycin.

Two hundred and twenty eight children (25%, 95% CI 23–28%) had a positive TPPA. The prevalence did not differ significantly between individuals who had and had not received treatment with azithromycin (24% vs 26%, p = 0.598). Thirty two children (3.5%, 95% CI 2.5–4.9%) had a positive TPPA and an RPR titre of ≥ 1:4; the prevalence of this differed significantly between individuals who had and had not received treatment with azithromycin (2.8% vs 6.6%, p = 0.015). 11 children (1.2%, 95% CI 0.06–2.2%) had a high titre positive RPR and this also differed significantly between individuals who had and had not received azithromycin (0.8% vs 2.7%, p = 0.046) (Fig 1). We collected lesion swabs from twenty individuals. Swabs could not be collected from eight ulcerative lesions as they were dry. No sample tested positive for *T*. *p* subsp. *pertenue*, but 7 swabs (35%) were positive for *H*. *ducreyi*.

Given the small number of individuals with dual sero-positivity these subjects were combined into a single group for the purpose of further analysis. People who had not taken azithromycin had higher odds of dual sero-positivity than those who had (OR = 2.49, 95% CI 1.2–5.2, p = 0.015), and after adjusting for confounding due to age, gender, and community of residence, the odds ratio was 3.8 (95% CI 1.8–8.5, p = 0.001) (Tables 2 and 3). Increasing age was associated with TPPA positivity, but no other variable was associated with dual sero-positivity.

**Table 2:** Risk factors for TPPA Positivity.

Variable	Indicative prevalence data	Unadjusted Odds Ratio	95% CI	p-value
Age*	5–10: 21%	1.1	1.1–1.1	<0.001
	11–14: 32%			
Male	Male: 27%	1.2	0.9–1.6	0.247
	Female: 24%			
Household size	≤5: 25%	1.0	0.8–1.4	0.836
	>5: 26%			
Reported taking	Yes: 26%	0.9	0.6–1.3	0.598
azithromycin	No: 24%			
Household MDA	Complete: 25%	0.8	0.5–1.4	0.472
	Incomplete: 25%			
	None: 28%	0.9	0.5–1.3	

*Risk associated with a 1 year increase in age

**Table 3:** Risk factors for dual sero-positivity.

Variable	Indicative prevalence data	Unadjusted Odds Ratio	95% CI	p-value
Age	5–10: 3%	1.1	0.9–1.2	0.291
	11–14: 4%			
Male	Male: 4%	1.4	0.7–2.8	0.394
	Female: 3%			
Household size	≤5: 4%	0.8	0.4–1.7	0.5980
	>5: 3%			
Reported taking azithromycin	Yes: 2.8%	2.5	1.2–5.2	0.015
	No: 6.7%			
Household MDA	Complete: 3.0%			0.097
	Incomplete: 3.2%	1.1	0.5–2.6	
	None: 6.8%	2.4	0.9–5.7	

**Fig 2:**
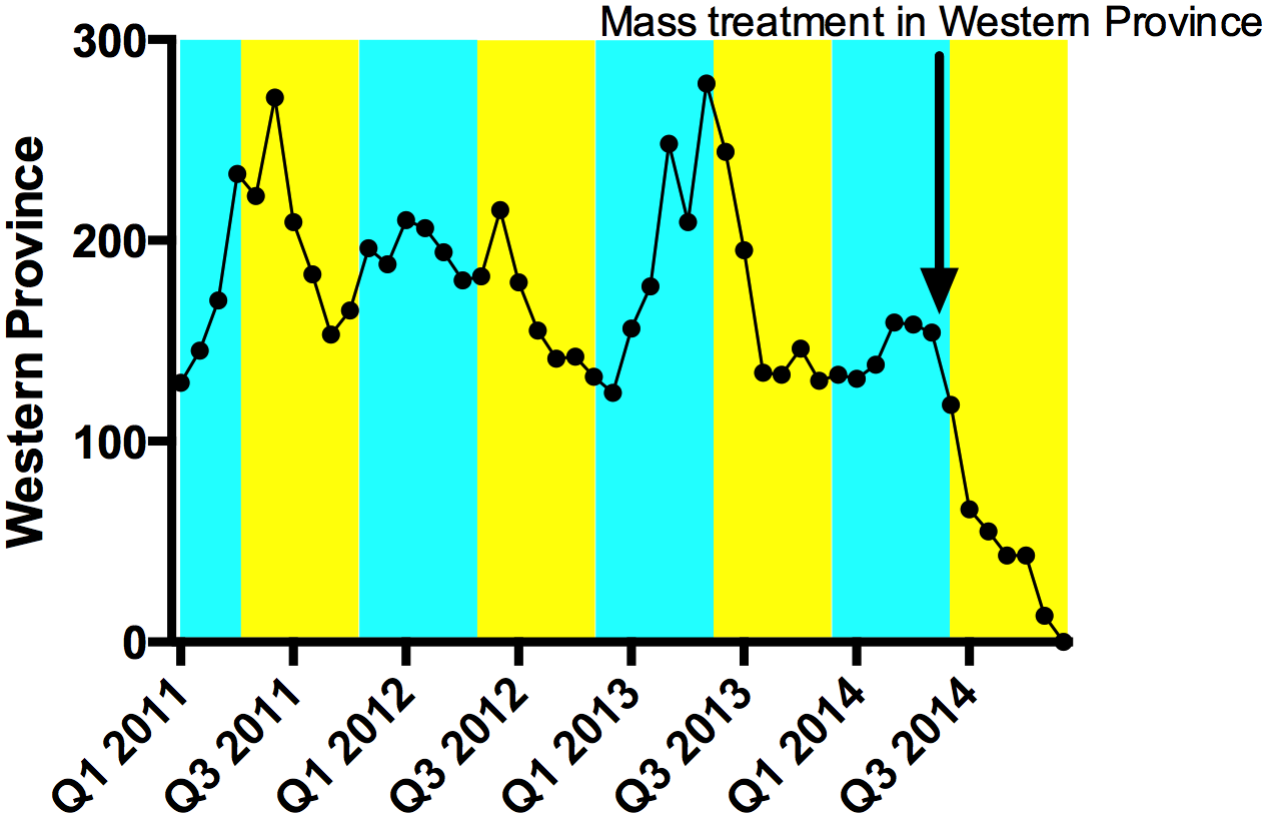
Yaws clinical case reporting in Western Province, 2011–2014. The rainy season is indicated in blue and the dry season in yellow. In the pre-mass treatment period there is evidence of seasonal variation in the incidence of yaws, which is well recognized. Following mass treatment there was a profound drop in the number of cases reported.

In the pre-mass treatment period (n = 36 months) the mean monthly number of cases of yaws reported by clinicians in the Western Province was 184. In the interrupted time series analysis, the number of cases was 183 in the dry season and 158 in the wet season (p = 0.440), and mass treatment was followed by a reduction in the mean number of cases reported per month of 101 case (relative reduction 57%, p = 0.044) (Fig 2).

## Discussion

In this study, a single round of community mass treatment with 20mg/kg azithromycin, given for trachoma elimination, resulted in a significant reduction in the prevalence of both active and latent yaws, from 1.5% and 20.2% pre-treatment[12], to 0.0% and 3.6% post-treatment (p = 0.002 and <0.001, respectively). The prevalence of infection declined both in individuals who had received treatment and in those who had not, suggesting that a single round of treatment may have reduced transmission, resulting in a population level benefit that extended to individuals who were not themselves treated. Consistent with this, the impact of azithromycin appeared particularly marked in reducing the prevalence of high-titre positive individuals, who are thought to drive transmission at community level. Our results are mirrored in the routine reporting data for incident yaws cases, which showed a profound drop following mass treatment with azithromycin. There was also a reduction in the prevalence of any ulcerative skin lesion since our previous survey (6.0% vs 3.1%, p = 0.004). Taken together, these data suggest that a single round of 20mg/kg azithromycin mass treatment given for trachoma may have interrupted yaws transmission, resulting in a reduction of both prevalent and incident yaws cases, and reducing the prevalence of skin lesions due to other bacteria.

The results of this study are concordant with recently published data from Papua New Guinea, which also demonstrated that a single round of azithromycin mass treatment, albeit at a higher dose of 30mg/kg, significantly reduced the prevalence of active and latent yaws[19]. In our study, effectiveness was demonstrated with a lower dose of azithromycin (20mg/kg), evidence that facilitates integration of yaws control into national trachoma elimination plans. The absence of any lesions which were positive by PCR for *T*. *p* subsp. *pertenue* is consistent with the marked effect seen on serological markers of infection. Our failure to detect treponemal DNA is somewhat reassuring in the context of the theoretical potential for lower dose azithromycin to select for macrolide resistance[20]. Integrated, synergistic control efforts are likely to result in increased efficiencies and decreased costs for programmes and ministries of health, which will be vital in helping countries achieve elimination targets by 2020.

In this population, individual level coverage with azithromycin was about 80%, which is in line with that commonly achieved by trachoma elimination programmes. Our findings suggest that an initial mass treatment round with high coverage can significantly reduce the burden of infection. Whether subsequent treatment would be best delivered through community mass treatment or the detection of cases and contacts remains unclear and should be studied further using both observational and modeling approaches. In view of the extremely low positive predictive value of clinical signs for the diagnosis of yaws seen here, the call for point of care serological tests to be made available within the health care system[21] must be redoubled, in order to strengthen surveillance and guide post-mass treatment case detection and treatment.

In trachoma control programmes in sub-Saharan Africa the use of height-based dosing algorithms commonly results in children receiving doses of azithromycin closer to 30mg/kg than 20mg/kg[22] which might make it difficult to detect meaningful differences in outcomes between the two dosing strategies. As there were limited anthropometric data to guide height-based dosing in the Pacific, weight-based dosing was used in the Solomon Islands, and children therefore received a dose as close as possible to 20mg/kg body weight, to a maximum of 1g. This study therefore provides the first prospective data supporting the effectiveness of lower dose azithromycin against yaws. This information is of particular value for countries where yaws and trachoma are co-endemic and which may therefore benefit from existing trachoma elimination activities.

The most notable limitation of this study is its observational nature. Whilst a randomized design may have been desirable, this would have be unethical, given the need to implement international guidelines for trachoma elimination[23], which mandate treatment of the whole population. A stepped-wedge design could have been considered[24] but may have been unethical, for the same reason. Follow-up in this study was limited to 6 months, and it is possible that longer observation would have revealed a more marked difference between the two groups. We relied on reported receipt of azithromycin, which may have introduced an element of recall bias. However it is likely that this would, in fact, have reduced any difference seen between individuals who did and did not receive azithromycin, and therefore would not affect the overall finding of our study. RPR titres normally fall rapidly in individuals successfully treated for yaws and, in the original randomized control trial of azithromycin conducted in Papua New Guinea[5], combined clinical and serological cure was 96% at 6 months. It seems likely therefore that we have observed the greater part of the effect that might be expected to be derived from azithromycin mass treatment.

Our findings support the roll out of mass treatment with azithromycin as an effective intervention for the simultaneous elimination of trachoma and yaws in co-endemic areas, and provide further observational data to recommend the WHO Morges strategy where yaws alone is endemic. The reduction in the prevalence of latent yaws following community mass treatment is a particularly important result, as a failure to adequately treat these individuals is thought to have contributed to the failure of previous yaws eradication efforts. Community mobilization, ongoing surveillance and lasting political support will be necessary to translate these findings into the ambitious goal of yaws eradication.

## Supporting Information

S1 Checklist Strobe checklist. (DOCX)

S1 File Supplementary data file. (XLS)
